# Hooked Up from a Distance: Charting Genome-Wide Long-Range Interaction Maps in Neural Cells Chromatin to Identify Novel Candidate Genes for Neurodevelopmental Disorders

**DOI:** 10.3390/ijms24021164

**Published:** 2023-01-06

**Authors:** Sara Mercurio, Giorgia Pozzolini, Roberta Baldi, Sara E. Barilà, Mattia Pitasi, Orazio Catona, Romina D’Aurizio, Silvia K. Nicolis

**Affiliations:** 1 Department of Biotechnology and Biosciences, University of Milano-Bicocca, piazza della Scienza 2, 20126 Milano, Italy; 2 Institute of Informatics and Telematics (IIT), National Research Council (CNR), 56124 Pisa, Italy

**Keywords:** enhancers, chromatin, long-range interactions, DNA sequence variants, neurodevelopmental disorders (NDD), CRISPR-Cas9, gene regulation

## Abstract

DNA sequence variants (single nucleotide polymorphisms or variants, SNPs/SNVs; copy number variants, CNVs) associated to neurodevelopmental disorders (NDD) and traits often map on putative transcriptional regulatory elements, including, in particular, enhancers. However, the genes controlled by these enhancers remain poorly defined. Traditionally, the activity of a given enhancer, and the effect of its possible alteration associated to the sequence variants, has been thought to influence the nearest gene promoter. However, the obtainment of genome-wide long-range interaction maps in neural cells chromatin challenged this view, showing that a given enhancer is very frequently not connected to the nearest promoter, but to a more distant one, skipping genes in between. In this Perspective, we review some recent papers, who generated long-range interaction maps (by HiC, RNApolII ChIA-PET, Capture-HiC, or PLACseq), and overlapped the identified long-range interacting DNA segments with DNA sequence variants associated to NDD (such as schizophrenia, bipolar disorder and autism) and traits (intelligence). This strategy allowed to attribute the function of enhancers, hosting the NDD-related sequence variants, to a connected gene promoter lying far away on the linear chromosome map. Some of these enhancer-connected genes had indeed been already identified as contributive to the diseases, by the identification of mutations within the gene’s protein-coding regions (exons), validating the approach. Significantly, however, the connected genes also include many genes that were not previously found mutated in their exons, pointing to novel candidate contributors to NDD and traits. Thus, long-range interaction maps, in combination with DNA variants detected in association with NDD, can be used as “pointers” to identify novel candidate disease-relevant genes. Functional manipulation of the long-range interaction network involving enhancers and promoters by CRISPR-Cas9-based approaches is beginning to probe for the functional significance of the identified interactions, and the enhancers and the genes involved, improving our understanding of neural development and its pathology.

## 1. Introduction

**Figure 1:**
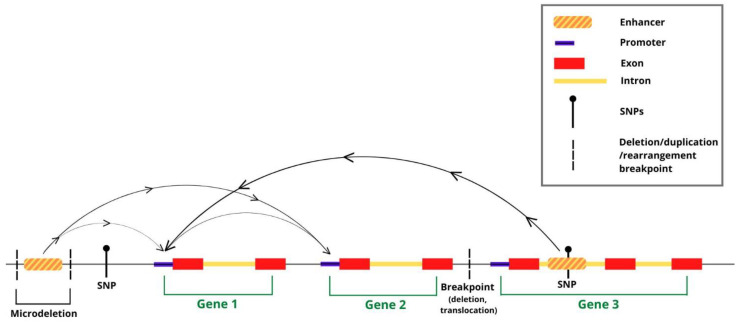
Long-range interactions in chromatin (arches) connect distant putative enhancers to gene promoters. Putative enhancers are shown, overlapping DNA sequence variants (SNPs; CNVs, such as deletions/duplications/rearrangements) associated to NDD. Interactions provide a way to associate variant-containing enhancers to distant gene promoters (arrows on the arches-connections), which they may regulate/misregulate in disease. Putative enhancers connected to more than one promoter, as well as promoter-promoter interactions, have also been detected (see text).

**Figure 2:**
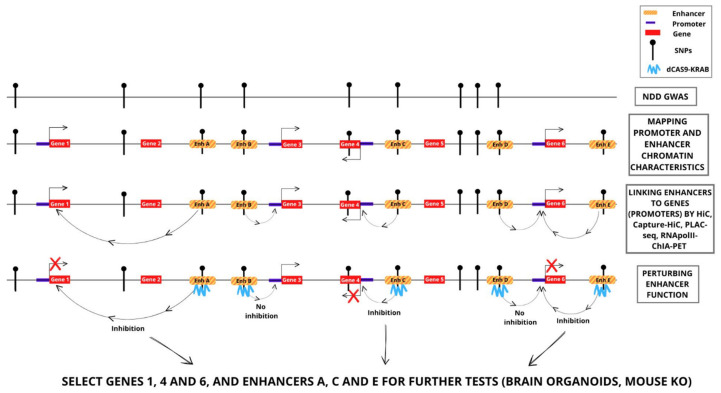
Identification and functional investigation of enhancer–gene promoter interactions involving DNA sequence variants (SNPs) associated to NDD. Examples of the strategies discussed in the text, and of possible results. Perturbation, by CRISPR-dCas9KRAB, of enhancers A, C and E inhibits the connected genes (genes 1, 4 and 6, respectively); perturbation of enhancers B and D does not inhibit the connected genes. Thus, genes 1, 4 and 6 are candidate NDD genes. Gene 4 was also previously suggested to be an NDD gene by polymorphic variation (SNP) within the gene, associated by GWAS to NDD.

Functional genomics approaches based on high-throughput sequencing (such as ChIPseq and ATACseq) allowed the mapping of hundreds of thousands of candidate enhancers within the human and mouse genome [1]. Enhancers are fundamental DNA sequences active in gene transcription, which they regulate by functional interaction with gene promoters; both enhancers and promoters interact with combinations of transcription factors, which, together with RNApolymerase II (RNApolII), regulate the transcriptional output of a given gene [1]. These sequences carry epigenetic “enhancer marks”, such as histone modifications and transcription factor binding sites (identified by ChIPseq assays), and “open” chromatin configuration (identified by ATACseq and related assays). Following the first genome-wide functional genomics investigations, it became immediately apparent that DNA sequence variants, previously associated to inherited disorders by genome-wide association studies (GWAS), which mostly lie within the non-coding genome, very frequently mapped onto, or very near to, these “epigenetic enhancers” at the genome-wide scale [2,3,4,5] (Figure 1 and Figure 2). This raised the hypothesis that the disease-associated sequence variants themselves, or closely linked mutations, may act by altering the activity of enhancers. However, which gene(s) are regulated by these enhancers, and possibly deregulated by associated mutations? The traditional view that an enhancer regulates the nearest gene promoter was substantially challenged, at the genome-wide level, by the experimental determination of genome-wide long-range interaction maps in the human and mouse genome. These maps in fact showed that epigenetic enhancers are very frequently (most frequently, at least in some cell types) physically and functionally connected to distant gene promoters, skipping genes in between, and enhancers within a genes’ intron can be connected to a promoter different from the one in whose intron they map (Figure 1) [1,6].

Here, we review a set of recent papers who experimentally determined long-range interaction maps in neural cells, overlapped them with datasets of DNA sequence variants associated to NDD, and validated some of the identified interactions by CRISPR-Cas9-based approaches, pointing to novel NDD-contributing genes.

## 2. Charting Long-Range Interactions in Chromatin

Long-range interaction maps identify physical association, in chromatin, between DNA sequences lying distant on the linear chromosome map. They are based on the “freezing” of the physical proximity by crosslinking of chromatin, followed by DNA fragmentation, ligation of the physically associated DNA fragments and high-throughput sequencing [7,8]. The studies we discuss here (Table 1) mapped interactions by different methods. Hi-C [8,9,10] theoretically makes it possible to map all interactions between every pair of DNA fragments in a single reaction. HiC gives a global view of 3D chromosomal topology, and revealed large-scale chromosomal structures such as A/B compartments [9] and topologically associated domains [11,12,13]. The global nature of HiC, however, results in extreme complexity of HiC libraries (i.e., all pairwise contacts between ~10^6^ and 10^8^ fragments in a mammalian genome, depending on the restriction enzyme used); this, in turn, requires an impractical sequencing depth to allow for robust and sensitive detection of individual chromosomal contacts [14]. RNApolII-Chromatin Interaction Analysis by Paired-End Tagging (ChIA-PET), and its variant RNApolII-in situ ChIA-PET [6,15,16], introduces a chromatin immunoprecipitation step with anti-RNApolII antibodies, prior to proximity ligation, thereby selecting for those interactions that are truly associated to gene transcription. Most connected enhancers detected by this method in brain-derived neural stem cells (NSC) (14/15 tested) behave as enhancers active in the brain in *in vivo* transgenesis assays in zebrafish and mouse [15]. HiC combined with targeted capture and sequencing (Capture HiC, CHiC) attempts to overcome the original HiC limitation by using thousands of sequence capture probes to enrich HiC material for interactions that involve, at least on one end, restriction fragments of interest (‘baits’or ‘viewpoints’), such as all annotated gene promoters [17,18]. Finally, in Proximity-Ligation-Assisted ChIPseq (PLAC-seq), an immunoprecipitation step with antibodies against H3K4me3, a histone modification found on active promoters, enriches for interactions involving transcribed gene promoters [19].

## 3. DNA Sequence Variants (SNVs; CNVs) Overlap with Connected Enhancers Identified by Genome-Wide Long-Range Interaction Maps, and Are Often “Associated” to NDD by GWAS Studies

DNA variants detected within genes, are often associated to functional abnormalities of the regulation of gene expression or of the encoded protein, which may be related to inherited monogenic or multifactorial disease [20,21]. The discovery of hundreds of thousands of variants mapping in non-coding DNA or putative enhancers has suggested that such variants might play a role in the regulation of the gene controlled by the enhancer connected to it, and possibly in the disease (Figure 2). To investigate this point, it is first essential: (i) to identify the connected gene; (ii) to determine whether the enhancer indeed regulates the connected gene (increases or decreases the activity of the gene); and (iii) to evaluate the activity of the variant versus the activity of the wild type enhancer. If such points are correctly demonstrated, it can hypothesized that the variant enhancer contributes to the disease by causing abnormal regulation of the connected gene, that can then be considered a candidate disease gene.

The studies reviewed below were performed using a variety of neural cell types, at different developmental stages, and identified candidate NDD disease genes overlapping between different studies, as well new genes revealed specifically in each individual work. This emphasizes the necessity, for the complex brain system, to investigate many different cell types and developmental times, to accurately cover the spectrum of candidate mutations.

Genome-wide informatic studies of DNA sequence variants, previously identified as associated (by GWAS) with NDD and traits, identified large numbers of epigenetic enhancers carrying DNA variants, and connected with gene promoters by interactions in neural cells (here termed “connected enhancers”) (Table 1; Figure 2). Most such studies were performed by HiC, or related variants of the technique.

Won et al. [22] overlapped enhancer–promoter chromatin contacts, identified by HiC in mid-gestation developing human cerebral cortex, with non-coding variants (SNPs) identified in schizophrenia (SCZ) by GWAS. This revealed multiple candidate SCZ risk genes and pathways. Several among these genes were also supported as candidate risk genes by independent analyses of expression quantitative trait loci (eQTL). Significantly, the study showed that most HiC-identified enhancers did not interact with adjacent genes, and that a relevant fraction of genes interacted with enhancers in a brain-specific manner, emphasizing the importance of identifying tissue-relevant chromatin interactions. Overall, contacts between enhancers and gene promoters revealed by HiC identified about 500 genes that were not adjacent to index SNPs, in agreement with the idea that linear chromosome organization per se does not capture the majority of regulatory interactions. These candidate SCZ genes were enriched for postsynaptic proteins, acetylcholine receptors, neuronal differentiation proteins and chromatin remodellers. Of note, they included various transcription factors, such as SOX2, FOXG1, EMX1, TBR1, SATB2, CUX2 and FOXP1.

Rajarajan et al. [23], in the framework of the PsychENCODE consortium work (see https://synapse.org (accessed on 1 January 2023)), mapped long-range interactions by HiC in neural progenitor cells (NPCs) derived from human induced pluripotent stem cell (hIPSC), and in neurons and astrocyte-like glia derived from NPC differentiation (Table 1). They overlapped their maps with SCZ risk loci. A total of 224 genes overlapped the risk loci themselves; 580 genes did not overlap themselves, with the risk loci, but were identified by their long-range interactions with distant epigenetic enhancers, overlapping the SCZ risk loci (240 in NPCs, 227 in neurons, 113 in astroglia). These genes (called “risk-locus connect”) expanded by up to 150% the current network of known genes overlapping risk sequences informed only by GWAS. The identified disease-related “connectome” was enriched in genes related to neuronal connectivity, synaptic plasticity and chromatin-associated proteins, including TFs [23].

Jung et al. [17] used capture-HiC to generate maps of long-range interactions in 27 human cell/tissue types. In the nervous system, the investigated cell types included neural progenitor cells, and some primary tissue types (e.g., hippocampus, dorsolateral prefrontal cortex). They subsequently overlapped their interaction maps with a catalog of variants identified by GWAS [24]. A significant portion of SNPs were found to map within putative cis-regulatory elements, i.e., elements carrying epigenetic features suggesting their active involvement in transcriptional regulation [1]. Notably, the interaction maps provided many more gene predictions of disease-associated genes than the nearest-neighbor-gene predictions alone. In fact, only about 8% of the putative target genes, that had been inferred from the interaction of their promoter with the variant-containing enhancer, were found to be the gene closest to the sequence variant (in agreement with Won et al.).

Nott et al. [25] used H3K4me3-PLAC-seq to generate interaction maps starting from human microglia, neurons and oligodendrocytes, isolated from resected cortical brain tissue from six individuals by fluorescence-activated cell sorting (FACS), using antibody-recognizing NEUN (neurons), OLIG2 (oligodendrocytes) and PU1 (microglia). They first determined the enrichment of genetic variants associated to specific NDD in cell-type-specific regulatory regions identified by ATAC-seq, H3K27Ac and H3K4me3 ChIPseq, and found a strong enrichment of heritability for variants within neuronal enhancers and promoters for brain function disorders and traits (including ASD, SCZ and BD). For this analysis, they used linkage disequilibrium score regression (LDSR) analysis of heritability, a method that separately quantifies the contribution of polygenic effects from that of different confounding factors (e.g., systematic differences in allele frequencies between subpopulations, unrelated to the disease) [26]. Notably, in Alzheimer’s disease (AD) SNP heritability was most highly enriched in microglia enhancers. These enhancers were found to be connected either to genes already directly identified as candidate disease genes by GWAS, or to “novel” genes not previously described as candidate disease genes. Interestingly, some of the microglia enhancers detected by these studies appear to have chromatin enhancer characteristics only in microglia, but not in other cell types.

Song et al. (2019) [27] mapped interactions within human neurons derived from hiPSCs, by promoter-capture HiC. They found ample overlaps with SNPs previously associated with brain disorders with a genetic contribution such as attention deficit hyperactivity disorder (ADHD), ASD, BD and SCZ (Table 1).

Song et al. [28] subsequently used H3K4me3-PLAC-seq to profile interactions from the germinal zone (GZ) and cortical plate (CP), microdissected from mid-gestation human cortex and sorted by FACS using appropriate of combinations antibodies against SOX2, PAX6, EOMES and SATB2 to isolate Radial Glia (RG), intermediate progenitors (IPC), excitatory neurons (eN) and interneurons (iN). This identified 35,552, 26,138, 29,104 and 22,598 interactions for RG, IPC, eN and iN, respectively. Song et al. overlapped their interacting enhancers/promoters with SNPs associated with seven brain disorders with genetic contribution: AD, ADHD, ASD, BD, intelligence quotient (IQ), major depressive disorder (MDD) and SCZ. The interacting sequences showed significant enrichment for all the traits except ASD and AD.

Bertolini et al. [15] used a different approach, i.e., RNApolII-ChIA-PET, to profile RNA-polII-mediated long-range interactions in the chromatin of ex vivo neural stem/progenitor cells (NSC) from the mouse neonatal forebrain, in the normal (wild-type) and in mice in which the SOX2 transcription factor-encoding gene (whose mutation causes NDD in humans) had been deleted in the developing nervous system via a Nestin-Cre transgene ([29], see also [30]). The comparison between the SOX2+ and SOX2- NSC interactions showed that SOX2 is essential to maintain the integrity of the 3D interactome, as well as the expression levels of a proportion of genes involved in SOX2-dependent interactions. Of note, enhancers and promoters of genes, known to cause NDD when mutated, were highly enriched in SOX2-bound interactions. The identified connected enhancers were mostly active (14/15 connected enhancers tested) in transgenic zebrafish and mouse, directing the expression of reporter genes to the developing forebrain; this indicated the ability of RNApolII ChIA-PET to identify functional enhancers active in the brain *in vivo*. Further, the vast majority of the connected enhancers, identified in NSC by RNApolII ChIA-PET, had a counterpart in epigenetic enhancers carrying active enhancer marks in the adult brain [31], as defined by overlap between connected RNApolII ChIA-PET enhancers and epigenetic enhancers (candidate cis-regulatory elements, cCREs) previously defined by Li et al. [32]. Out of about 10,000 mouse interactions involving epigenetic enhancers (on one “anchor”) and gene promoters (on the other “anchor”), about 7500 were conserved in humans [31]. For these interactions, both the enhancer and promoter regions could be re-mapped onto a syntenic region of the human genome; in addition, most human enhancers and promoters carried epigenetic marks of activity (H3K27Ac; H3K4me1) also in human neural cells, and were connected in interaction maps [31]. The identified connected enhancers were subsequently overlapped with DNA sequence variants associated to NDD, of both the SNV and CNV type [31]. The overlap with CNV identified in ASD patients showed that microdeletions could overlap with enhancers, connected, in turn, to distant genes (not involved, themselves, in the deletion). In other cases, CNVs involving longer regions separated a connected enhancer from its target gene; the gene was not involved itself in the CNV, but may be deregulated by the loss of regulatory interactions. Further, many SNV, previously associated with SCZ, bipolar disorder (BD) and intelligence, were found to overlap with connected enhancers identified by ChIA-PET; while some were connected to genes, already identified as NDD contributors by mutations in the gene’s protein coding region, others were connected to genes active in the developing nervous system and in neurons, but not previously involved in NDD pathogenesis. Significantly, while some of these genes had already emerged in the studies discussed above, mapping interactions with HiC, about half of the genes were newly identified by the ChIA-PET maps [31], pointing to the usefulness of different, complementary mapping methods for a thorough charting of chromatin interactions relevant for NDD.

**Table 1:** Studies linking NDD to enhancer variants and their interacting genes.

Study	Interactions Mapping Method	NDD/Trait Investigated	Enhancer Validation (Transgenesis)	Targeted CRISPR-Cas9 Validation of E-P Connections	Cell Source
Won et al., Nature 2016, 538:523 [22]	HiC	SCZ	NA	1 (FOXG1-connected)	Human fetal brain
Rajarajan et al. PsychENCODE, Science 2018, 362: 1269 [23]	HiC	SCZ	NA	3 (ASCL1, EFNB1, or MATR3-connected in NPC)	Human iPSC-derived NPCs, neurons, glia
Jung et al. Nat Genetics 2019, 51:1442-49 [17]	Capture HiC	Brain-related disorders in SNP general catalog (Welter et al., NAR 2014 42: D1001-D1006, [24])	NA	5 (NT5DC2-connected in lymphoblastoid cells)+1 (TMED-4 connected)+1 (enhancer-like connected promoter connected to NCKIPSD)	Dorsolateral prefrontal human cortex, hippocampus
Nott et al. Science 2019, 366:1134 [25]	PLAC-seq	AD	NA	1 (BIN1-connected, del. effective in microglia)	Microglia, neurons, oligodendrocytes FACSed from resected human cortical brain tissue
Song et al., Nat Gen 2019, 51:1252 [27]	Promoter capture HiC	AD, ADHD, BD, ASD, ALS, EP, FTD, MP, PD, SCZ, UD	2 CDK5RAP3-connected enhancers active in mouse brain	2/2 (CDK5RAP3-connected in excitatory neurons)	Human iPSC-derived neurons; astrocytes
Song et al., Nature 2020, 587:664 [26]	PLAC-seq	AD, ADHD, BD, IQ, ASD, BD, MDD, SCZ	NA	3/7 (ADRA2-connected)+3 (GPX3-connected)+3 (IDH1-connected in RG and exc. neurons)	RG, IPC, eN, iN FACSed from GZ and CP from mid-gestation human cortex
Bertolini et al., Cell Stem Cell 2019, 24:462-476 [15]; D’Aurizio et al., IJMS 2022 [27]	RNApolII-ChIA-PET	SCZ, BD, intelligence; CNVs associated to ASD, ADHD, SCZ, OCD, neurocognitive disorders	14/15 connected enhancers active in zebrafish and/or mouse brain	NA	Forebrain-derived NSC (mouse)

Abbreviations for disease names in Table 1 column 3 (NDD/Trait Investigated): SCZ, schizophrenia; AD, Alzheimer’s disease; ADHD, Attention deficit hyperactivity disorder; ASD, Autism spectrum disorder; ALS, Amyotrophic lateral sclerosis; BP, Bipolar disorder; EP, Epilepsy; FTD, Frontotemporal dementia; MP, Mental process; PD, Parkinson’s disease; UD, Unipolar depression; OCD, Obsessive-compulsive disorder; MDD, Major depressive disorder.

## 4. CRISPR-Cas9-Mediated Genome Editing Indicates That Some of the Connected NDD-Relevant Enhancers Identified by Interaction Maps Regulate Expression of the Connected Gene

Once an enhancer carrying a variant hypothesized to contribute to NDD is detected, it is necessary to show that the enhancer is able to regulate the expression of the connected genes (Figure 2). This is indeed a major problem if one is to evaluate large numbers of candidate enhancers, as a laborious methodology has to be developed. Further, it is to be emphasized that, while the demonstration of an effect of the studied enhancer on the connected promoter is greatly encouraging, this in no way proves that the variant detected in a given patient can in fact cause the disease. To address the first problem, several groups devised techniques to simultaneously inhibit (or activate) large numbers of different enhancers, to then go on to evaluate the activities of the connected genes.

## 5. Single-Enhancer Modulations

Single-enhancer modulation experiments (Figure 2) were performed by the Authors whose works have been already commented above, and that are reported in Table 1.

Initially, Won et al. [22] performed CRISPR-Cas9-based targeted enhancer deletion or epigenetic manipulation, in human neural progenitors, of a putative enhancer hosting a credible SCZ-associated SNP, connected to the FOXG1 gene, lying 760 kb away from the SCZ-associated region. Enhancer deletion significantly reduced the expression of FOXG1 (by 20–25%), but not that of the nearby PRKD1 gene, as evaluated by qRT-PCR analysis. Further, the SNP (a T in the risk allele replacing a G) reduced the activity of the enhancer in a reporter assay in transfected cells by a similar percentage. The data are in agreement with the regulation of FOXG1 by the connected enhancer overlapping the SCZ-associated SNP.

Rajarajan et al. [23] used epigenomic editing with nuclease-defective Cas9 (dCas9) fused to transcriptional activator domains (VP64 or VPR), to test for the relevance of risk SNP-containing epigenetic enhancers onto the expression of the connected genes (ASCL1, 225 kb away; EFNB1, 335 kb away; MATR3, 695 kb away; SOX2, 5–600 kb away) in neural progenitor cells (NPCs). Significant gene expression changes (fold-changes in the order of 1.5–3 times from baseline, represented by negative control sgRNA) were observed for two of these genes, ASCL1 and EFNB1, but not for MATR3 nor SOX2; intriguingly, gene expression was decreased, though the targeted manipulation involved a dCas9-associated activator [23]. Subsequent mutagenesis (deletion), by Cas9 nuclease targeted by sgRNAs to a 138 bp sequence within the MATR3-connected enhancer, produced a significant increase in the connected MATR3 gene expression; similarly, targeted mutagenesis (deletion) of enhancers, overlapping credible SCZ-associated SNPs and connected to the ASCL1, EFNB1 and EP300 genes, significantly increased expression of the connected genes. Similar targeting of four credible SCZ-associated SNPs upstream of the clustered PCDH locus significantly decreased levels (by −50–60%) of PCDHA8 and PCDHA10 (two of the genes whose expression increased with the dosage of a risk SNP in postmortem brain).

Jung et al. [17] used CRISPR-Cas9 to validate the function of connected, “enhancer–like” promoters, which they had found connected in dorsolateral prefrontal cortex by promoter-capture HiC. They deleted the promoter of the ARIH2OS gene in human ES cells (hESC), which resulted in marked downregulation of the gene controlled by the connected gene promoter, NCKIPSD. Similarly, targeted deletion of the EIF4G1 promoter resulted in downregulation of the connected gene promoter ABCF3. It was noted that, in these cases, the gene downstream to the distal, “enhancer-like” promoter was not expressed, but carried active chromatin marks. Further, seven GWAS-associated variants were deleted by CRISPR-Cas9 mutagenesis in a lymphoblastoid cell line; for five out of seven tested elements, genetic perturbation led to downregulation of the predicted, connected target gene NT5DC2 by qRT-PCR.

Nott et al. [25] functionally investigated the BIN1 microglia-specific connected enhancer, which they found to be PLAC-linked to the BIN1 promoter (about 40,000 bp away), by CRISPR/Cas9-mediated deletion of a 363 bp region in two human pluripotent cell lines (PSC). Following targeted enhancer deletion, cells were differentiated into microglia, neurons and astrocytes. BIN1 expression was nearly absent in enhancer-deleted microglia, whereas expression in undifferentiated PSC, and in differentiated neurons and astrocytes, was equivalent to that in non-deleted cells. The observation that the most significant GWAS allele associated with BIN1 resides in a microglia-specific enhancer provides a rationale for further study of BIN1 function in this cell type.

Bertolini et al. [15] and D’Aurizio et al. [31] did not report a functional validation of the NDD-related interactions by targeted manipulation; however, recent initial work in our laboratory indicated that epigenetic repression, by dCas9KRAB, of two enhancers connected to the Olig2 promoter, one of which very close to a GWAS-identified SNV (associated to “educational attainment”), led to downregulation of the connected gene, pointing to a functional relevance of the identified interaction in NSC (unpublished). For a third connected enhancer, a smaller extent of downregulation was observed, that did not reach statistical significance. The extent of downregulation of the connected gene (maximum 50%) was always smaller than that observed with guideRNAs targeting the gene promoter (about 80–90%), in agreement with other published studies (Table 1) [33], suggesting that each connected enhancer accounts for only a part of the gene promoter activity.

Song et al. (2019) [27] functionally validated two enhancers, connected over 40 kb of distance to the promoter of the CDK5RAP3 gene, a regulator of the CDK5 gene, playing functional roles in neuronal development and controlling proliferation of non-neuronal cells. Targeted deletion of the first enhancer significantly reduced expression of the connected gene. Interestingly, targeting of the second enhancer in iPSC caused cell death, shortly after introduction of the sgRNA into cells. Lethality was circumvented by using CRISPR interference (CRISPRi) to epigenetically reduce the activity of enhancers 1 and 2 together, as well as of a third enhancer connected to CDKRAP3 in non-neuronal cell types. Targeting of enhancers 1 and 2, but not 3, led to significant downregulation of CDKRAP3, without influencing the expression of nearby genes.

Song et al. [28] validated some of their interactions by dCas9-KRAB-mediated modulation of connected enhancers in primary cells, developing CRISPR-view, a method allowing quantitative readout of transcriptional alterations at the single cell level via intronic probes. They validated four regions interacting with the GPX3 gene promoter; dCas9-KRAB-mediated silencing of these regions led to significant downregulation of the connected GPX3 gene. Further, the silencing of three regions interacting with the IDH1 promoter in RG and eN resulted in significant downregulation of their expression in the respective cell types [28].

Overall, these data indicate that a few connected enhancers, identified by various genome-wide interaction mapping protocols and overlapping with NDD-associated sequence variants, are indeed relevant for the regulation of the connected gene (Table 1). On the other hand, no explicit mention is made in most studies of how many such enhancers were challenged by genome editing, and how many scored negative for a role in the regulation of the connected gene. Given the existence of “shadow enhancers”, demonstrating enhancer redundancy in various developmentally relevant situations [34], a joint discussion of negative, together with positive, results might give further insight into the mechanisms of NDD-related enhancer function.

## 6. Multiple Enhancer Modulation Studies

While many enhancers have been identified, that contain potentially NDD-relevant variants, their functional study has so far been “low-throughput”, investigating one enhancer at a time, as indicated above. The targeting of many enhancers at one time, followed by single cell transcriptome analysis as a readout for gene expression changes [35], was originally developed in hematopoietic cells, and it may be adapted for the purpose of a multiplex enhancer study also in human neural cells.

In this work, the authors introduced stable dCAS9-KRAB lentiviruses to target 5920 different candidate enhancers, to affect the activity of each of these enhancers; on average, each cell was transduced by a combination of 15+/−11 guideRNAs-encoding viruses. The readout of this experiment was by RNA sequencing of single cells sorted by FACS, leading to the identification of 664 genes down-regulated by perturbation (downregulation) of the connected enhancer. A similar approach (CRISPRi-FlowFISH), again using dCAS9-KRAB [36,37,38], targets many putative enhancers with different guideRNAs in a pooled screen, followed by expression study of the gene(s) of interest: to this aim, fluorescence in situ hybridization (FISH) is used to quantitatively label single cells according to the expression of the mRNA(s) under investigation. Cells are then FACSed into six bins based on mRNA(s) of interest expression level, and high-throughput sequencing then determines the frequency of guideRNAs from each bin. Finally, the relative abundance of guideRNAs in each bin allows to compute the effects of the different guideRNAs on the expression of the investigated gene. This approach was useful for a vast study of disease-related GWAS variants in many cells and tissues, including brain cells [38]. In the latter study, the prediction of functional enhancer–gene connections was based on an Activity-By-Contact (“ABC”) model [37,38], that takes into consideration Activity (evaluated as measurement of chromatin accessibility (ATAC-seq or DNAse-seq), H3K24Ac histone modification (by ChIPseq) and chromatin Connectivity (HiC). The ABC model was shown to perform well in predicting (in silico) functional enhancer—promoter connections, as evaluated by CRISPRCas [36,37,38]. Together, these methodologies strengthen each other, allowing further insight into a potential role of non-coding variants in disease.

## 7. Does a GWAS Variant Detected within an Enhancer Connected to a Gene Identify a Role of the Connected Gene in the Disease?

The answer to this question is, in principle, no; it is, in fact, necessary, to show that this variant affects, in a suitable cellular/organ system, the activity of the connected gene. However, it is worth mentioning that, in some of the previously discussed papers, an enhancer carrying a GWAS variant was shown to be connected to a gene already identified as a candidate gene, for the same or a similar disease, by the detection, in some patients, of GWAS variants within the gene itself. These observations corroborate each other in suggesting the identification of the gene as a disease gene. Appropriate studies in cellular or organ system should then be encouraged by this type of finding.

Recently, in an attempt to compare many different regulatory sequence variants for their effect on gene activity levels, [39] used a massively parallel reporter assay (MPRA), coupled to CRISPR-based validation, to screen noncoding variants associated to AD and to progressive supranuclear palsy (PSP). Both alleles (wild-type and variant) of each regulatory sequence under study, were cloned into a reporter GFP expression vector, that had been differentially barcoded, downstream to the GFP sequence, to distinguish the transcript driven by the wild-type regulatory element from the transcript driven by the variant one. A library of over 5000 constructs was screened. After transfection into HEK293T cells, the activity of each allele was quantified by sequencing of the transcripts with the associated barcodes. Overall, 320 functional regulatory variants (frVars) across 27 loci were found to have a significant difference in expression between alleles, and were also enriched in epigenetic enhancer marks in human brain tissue. Forty-two such variants were selected for functional validation by CRISPR-based targeted perturbation. The candidate gene to be assayed in relation to a given variant was chosen based on previously reported eQTL or chromatin interaction data; overall, 19 frVars at 11 GWAS loci were validated in a pooled CRISPRi screen, or by targeted excision, in neurons, astrocytes or microglia derived from hIPSC. The results pointed to genes already previously identified as players in AD, as well as to previously unidentified risk genes for AD. Notably the identification of “active” variant alleles within functional regulatory variants pointed to the involvement of different classes of genes between AD and PSP. In particular, PSP is characterized by the disruption, due to the variant, of transcription factors-binding sites for SP1 or for SP1-interacting factors such as ARNT and TP63. This observation defines a pattern of regulatory interactions of the disrupted variants with target genes in *cis* within 10 kb or in trans, at greater distances.

The authors propose a model for combinatorial effects of common genetic risk. A transcription factor network converging on SP1 in PSP can be interpreted to suggest that common genetic variants may act on multiple binding sites for a given transcription factor (for example SP1 in PSP) to disrupt critical cell-type specific polygenic transcriptional programs.

## 8. Perspectives: Using Brain Organoids to Define the Effects of Variants during Early or Later Development In Vitro

Ultimately, the “knock-in” of the supposed pathogenic variants in the enhancers, now made possible by CRISPR-Cas9-mediated genome editing [40,41], will make it possible to clarify their role, in the context of an otherwise homogeneous genetic background. The study of alterations in gene expression, as well as their effect on neurogenesis, will be greatly helped by the development of protocols for the differentiation of human pluripotent stem cells into brain organoids in vitro, recapitulating important human-specific aspects of brain development [42].

The study of the genes, newly identified via their interactions with NDD-variant-containing enhancers, will equally benefit from brain organoid studies, complementing mouse models in the investigation of gene function. The possibility offered by CRISPR-Cas9 to perform targeted mutagenesis in human cells will allow to generate human cellular models to investigate the function of these new NDD-relevant genes.

So far, many reported functional enhancer manipulations involved the study of just one connected gene by RT-PCR. However, often enhancers are connected to more than one promoter, and promoters are connected among them, sometimes forming dense connectivity hubs [6]. The wider investigation in changes in gene expression in the genes within these hub regions will shed light on the possible co-regulation of genes within these hubs, and the possible consequences of mutations affecting regulatory elements. Indeed, it was shown that CRISPR-Cas9-mediated of a promoter can lead to the rewiring of enhancer–promoter connectivity, affecting different gene promoters [43].

Finally, both novel genes and enhancers, causally involved in NDD, may become the target of therapy approaches. While gene products can be functionally modulated by specific drugs, enhancers themselves have, recently, been the direct target of pharmacological manipulation, with a therapeutic effect, in thalassemia and sickle cell disease [44]. Enhancer-targeting [2] may involve drugs targeting transcription factors relevant for enhancer regulation [45,46], or CRISPR-Cas9 system components themselves. In the nervous system, CRISPR-Cas9 nanocomplexes were delivered in vivo to the mouse hippocampus, and were effective in targeting Bace1 in postmitotic neurons of the adult mouse brain, suppressing amyloid beta-associated pathologies in mouse models of AD [47].

In conclusion, the novel understanding of NDD-relevant gene regulation made possible by genome-wide interaction maps, coupled to CRISPR-Cas9-mediated targeted mutagenesis and epigenetic manipulation, will shed new light on the mechanisms of disease, and generate novel perspectives for therapy approaches.

## Data Availability

Links to publicly archived datasets analyzed are provided in the text. or in the papers discussed in this Perspective.
